# Small Bowel Obstruction Linked to Meckel's Diverticulum: A Rare Case

**DOI:** 10.7759/cureus.75731

**Published:** 2024-12-15

**Authors:** Fábio Viveiros, Nereida Monteiro, Inês Arnaud, Cláudia Lima, Rui Escaleira

**Affiliations:** 1 General Surgery, Local Health Unit of Alto Minho, Viana do Castelo, PRT; 2 Internal Medicine, Local Health Unit of Alto Minho, Ponte de Lima, PRT

**Keywords:** gastrointestinal obstruction, meckel's diverticulum, small bowel bleeding, small bowel resection, small bowel surgery

## Abstract

Meckel’s diverticulum (MD) is the most common congenital anomaly of the gastrointestinal tract, arising from incomplete obliteration of the vitelline duct. MD contains all layers of the intestinal wall and often remains asymptomatic. Gastrointestinal bleeding, bowel obstruction, and acute diverticulitis occur in a few cases. Risk factors for symptomatic MD include male sex, age < 50 years, diverticula > 2 cm, and the presence of ectopic tissue. We present a case report of a 61-year-old male presenting with acute abdominal pain and signs of small bowel obstruction. CT imaging revealed intestinal obstruction. The patient underwent an exploratory laparotomy, and an internal hernia caused by an MD adherent to the retroperitoneum was found. A segmental small bowel resection with ileo-ileal anastomosis was performed. Histological examination confirmed MD with ectopic gastric tissue. Management of MD should be guided by patient age, diverticulum characteristics, and histological features. This case highlights MD as a potential etiology for acute small bowel obstruction in patients without prior abdominal surgeries. Clinicians must maintain a high index of suspicion for MD in atypical presentations and tailor management to individual risk factors and intraoperative findings.

## Introduction

Meckel’s diverticulum (MD) is the most prevalent congenital anomaly of the gastrointestinal tract, occurring from incomplete obliteration of the vitelline duct during embryonic development. This anomaly forms a true diverticulum containing all layers of the intestinal wall and typically arises from the antimesenteric border of the distal ileum [1,2]. It represents a persistent remnant of the omphalomesenteric duct, which usually occurs between the fifth and sixth weeks of gestation. Failure to obliterate this duct can lead to various anomalies, including fistulas, cysts, and fibrous bands, although the diverticulum itself is the most common manifestation [3,4].

The prevalence of MD in the general population ranges from 0.3% to 2.9% [5]. MD has a male-to-female ratio of 2:1 and normally is found within 60 cm of the ileocecal valve [6,7]. Complications occur in 2%-4% of cases, predominantly in younger individuals [4].

MD is often clinically silent; when symptomatic, it usually begins with gastrointestinal bleeding or acute abdominal symptoms related to bowel obstruction, perforation, or diverticulitis [8,9].

Ectopic tissue is found in 12%-44% of symptomatic patients, with gastric heterotopia being the most frequent, followed by pancreatic and colonic tissue. The likelihood of symptomatic MD diminishes with age, but the risk persists across a lifetime. Risk factors for developing symptoms include age < 50 years, male sex, diverticula > 2 cm in length, and histologically abnormal tissue, which guides decisions on surgical resection [8].

Here, we present a case report of a small bowel obstruction caused by MD, followed by a review of the literature on this subject.

## Case presentation

A 61-year-old male presented to the surgical emergency room, complaining of abdominal pain associated with nausea and vomiting, which began three hours prior to arrival. The pain was diffuse, affecting the entire abdomen, and was not radiating. The abdominal pain was associated with nausea, and the patient experienced two episodes of alimentary vomiting. The patient also noted an absence of bowel movements (including gas) over the past three days but denied experiencing diarrhea, fever, or other associated symptoms.

Regarding his medical history, the patient had a known history of arterial hypertension and dyslipidemia, both managed with tailored pharmacological therapy, including an angiotensin-converting enzyme (ACE) inhibitor and a statin. His surgical history was unremarkable, with no previous abdominal surgeries or other surgical interventions.

Upon examination, the patient presented with a body temperature of 36.9 °C, exhibiting normal blood pressure (126/86 mmHg). His heart rate was 97 beats per minute. He was awake, alert, and exhibited a normal respiratory rate. Abdominal examination revealed tenderness across the entire abdomen, particularly pronounced in the right upper and lower quadrants. However, there were no signs of guarding or peritoneal irritation. Auscultation of the abdomen identified high-pitched bowel sounds.

Initial blood tests upon admission showed a hemoglobin level of 12.6 g/dL, an elevated white blood cell count of 17,450/ mm³ with 70% neutrophils, and a platelet count of 270,000/mm³. The C-reactive protein (CRP) level was elevated at 13.6 mg/dL, and serum lactate was slightly raised at 2.8 mmol/L. Other laboratory parameters, including coagulation biomarkers, were within normal limits (Table 1).

**Table 1:** Blood results of the patient on admission

Blood Test	Level Admission	Normal Range	Unit
Haemoglobin (Hb)	12.6	11.5-16.5	g/dL
White cell count	17.45 x 10^9^	4.0 - 11.0	10^9^/L
Platelets	270 x 10^9^	150-450	10^9^/L
Urea	6.2	2.5 – 7.8	mmol/L
Creatinine	90	50-98	umol/L
Alkaline phosphatase (ALP)	43	30 - 130	IU/L
Alanine transaminase (ALT)	14	0-55	IU/L
C-reactive protein	13.6	0.0 – 5.0	mg/dL
Serum lactate	2.8	0.0-2.0	mmol/L

**Figure 1:**
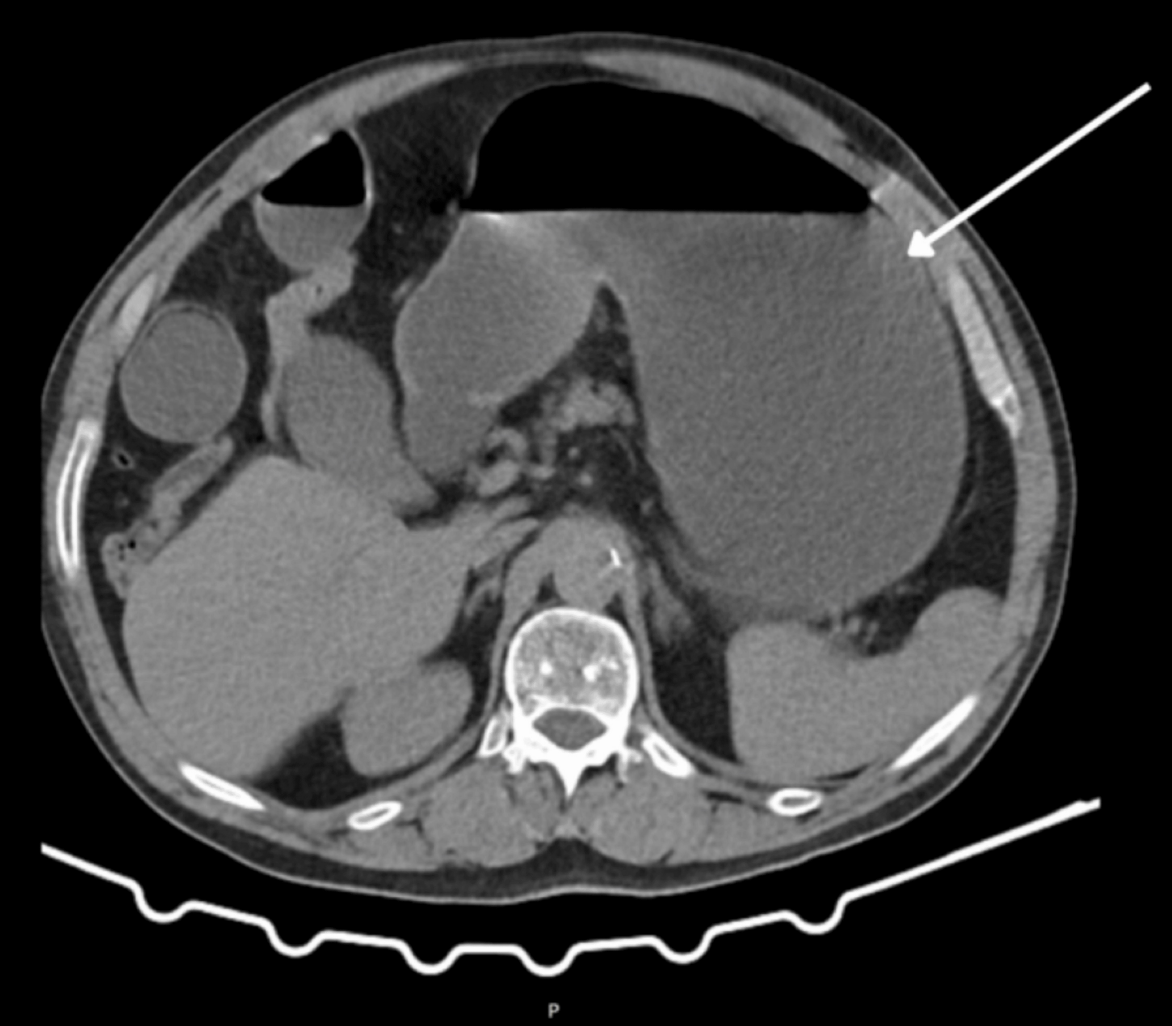
CT scan image - axial section This CT Scan shows gastric distention (arrow).

**Figure 2:**
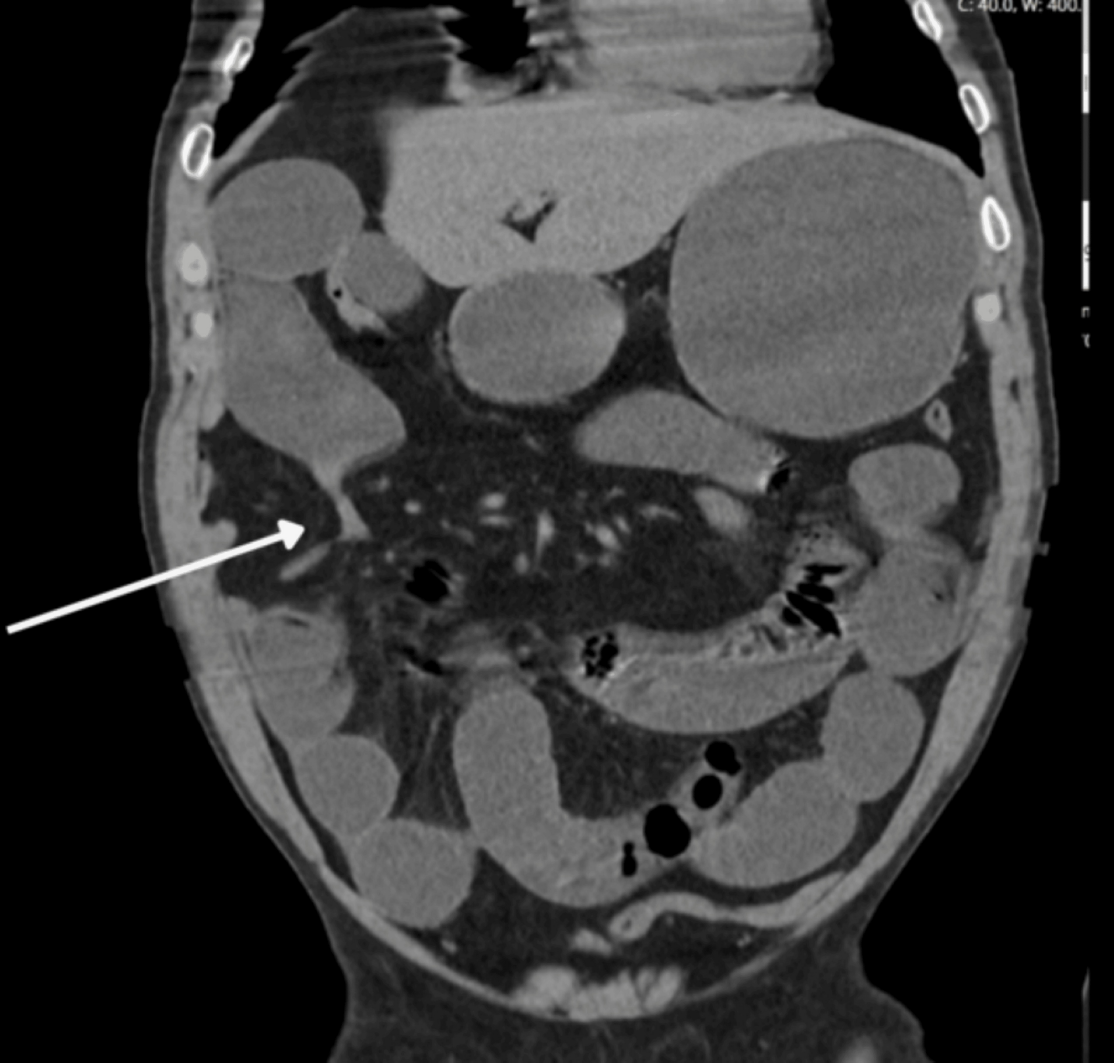
CT Scan Image - Coronal Section This image shows an abrupt narrowing of the small bowel lumen (arrow)

The abdominal CT scan (Figures 1-2) revealed signs of intestinal obstruction, characterized by distension of the stomach and small intestine extending to the right flank, where an abrupt narrowing of the lumen was noted. A loop was identified between the hepatic flexure or transverse colon and the abdominal wall, findings suggestive of obstruction potentially caused by adhesions or an internal hernia (transomental). No evidence of intestinal pneumatosis, pneumoperitoneum, collections, or free intraperitoneal fluid was observed.

Initial management included intravenous fluids, analgesia, and nasogastric tube was placed and the patient was kept nil by mouth (NPO). These measures were not very effective.

**Figure 3:**
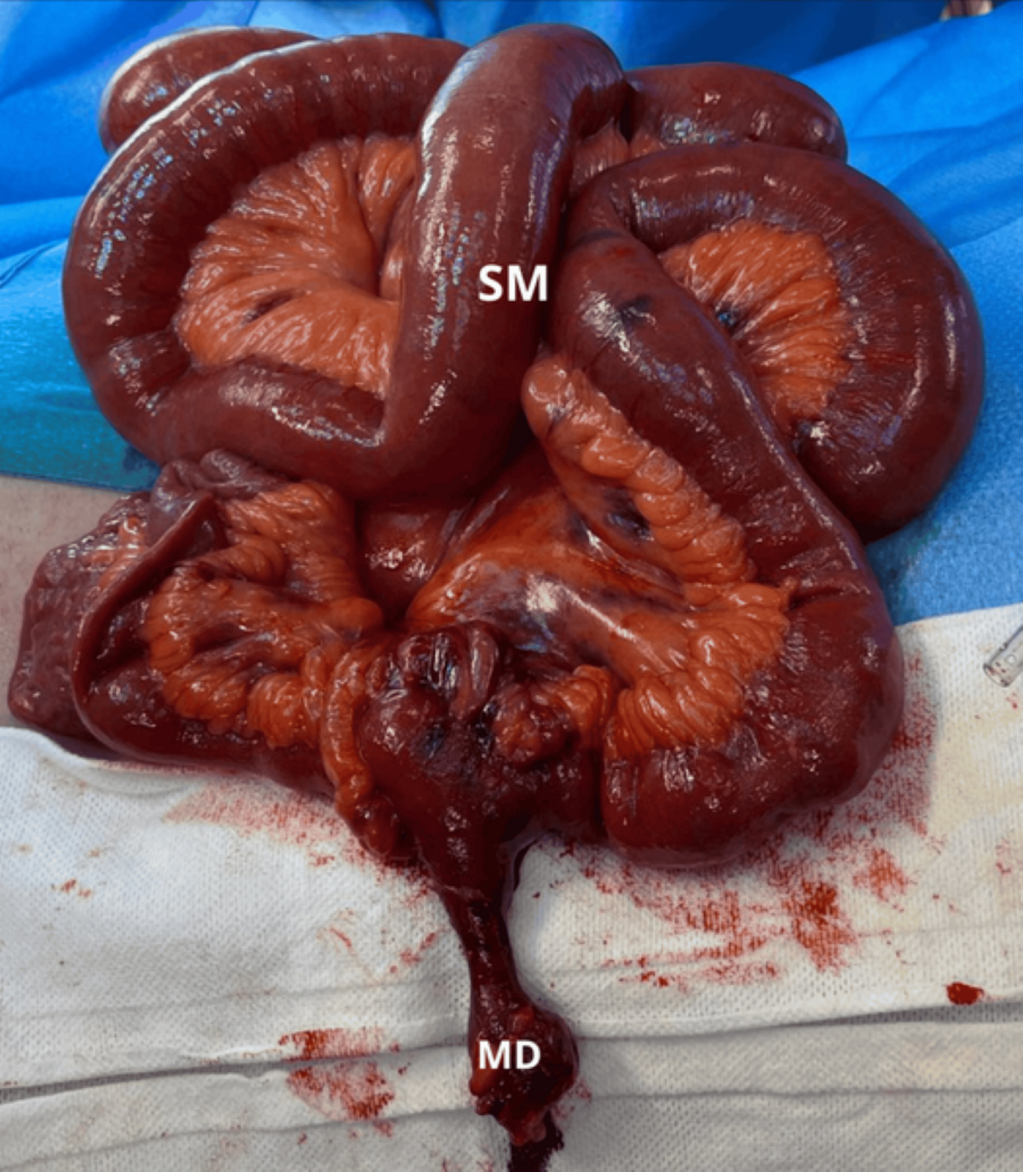
Intraoperative Gross Examination of the Small Bowel after reducing of the Internal Hernia In this image it can be seen the Meckel's Diverticulum (MD) along with the remaining small bowel (SM)

**Figure 4:**
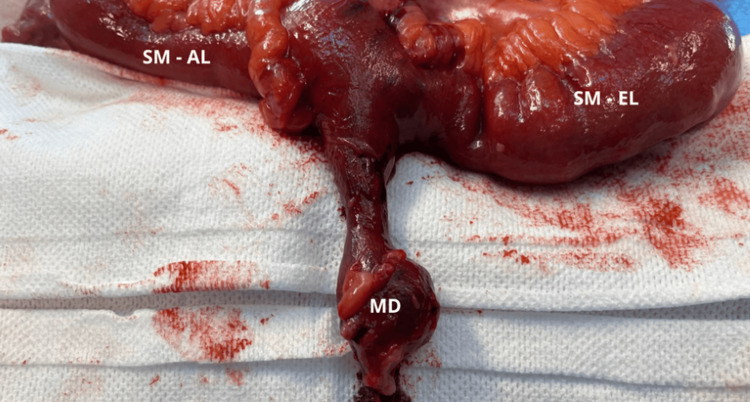
Intraoperative Gross Examination of the Meckel's Diverticulum (MD) before resection This image provides a close-up view of MD, along with the afferent limb (SM-AL) and efferent limb (SM-EL) of the small bowel

Therefore the patient underwent an exploratory laparotomy. Intraoperatively, and internal hernia originating from a Meckel’s diverticulum adherent to the retroperitoneum was identified, causing small bowel stenosis approximately 25 cm from the ileocecal valve. There were no signs of peritonitis. After reduction of the hernia, the segmental of small bowel containing the Meckel’s diverticulum appeared ischemic. Consequently, a segmental enterectomy was performed, followed by a side-to-side anisoperistaltic ileo-ileal mechanical anastomosis (Figure 3, and 4).

The post-operative period was unremarkable for complications and the patient underwent an uneventful recovery, and he was discharged after seven days. No long-term complications were acknowledged.

**Figure 5:**
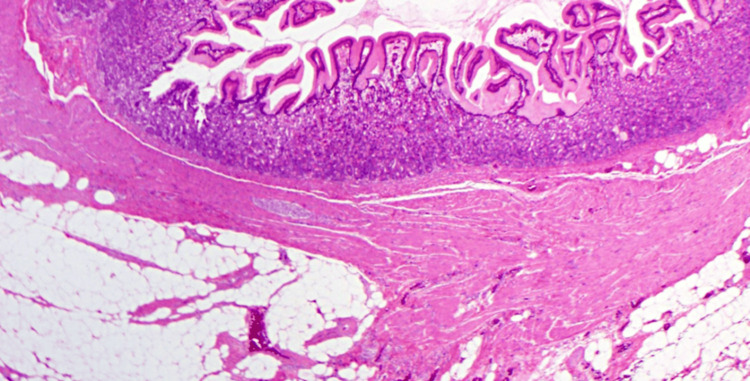
Histological Features of Meckel's Diverticulum (MD) Histological Features of MD with ectopic gastric tissue

The histological examination showed findings consistent with a Meckel’s diverticulum, in which ectopic gastric tissue was identified, with no other significant alterations (Figure 5).

## Discussion

It is estimated that MD affects approximately 2% of the general population. However, determining the exact prevalence is challenging, as many individuals with this condition remain asymptomatic [5,10].

To understand the possible complications of MD, it is necessary to understand its embryological features. In early embryonic development, the midgut connects to the yolk sac via the omphalomesenteric (vitelline) duct, which provides nutrition until the placenta takes over between the fifth and sixth weeks of gestation. If the duct fails to obliterate, anomalies such as MD may occur, often retaining the midgut mesentery blood supply [3,11]. Associated abnormalities include fistulas between the ileum and umbilicus, leading to bilious discharge, umbilical sinuses, vitelline cysts, or fibrous cords that can entangle bowel loops and cause obstruction. MD may also contain heterotopic tissues, commonly gastric or pancreatic, due to the pluripotent nature of the duct’s lining cells [12,13].

While most individuals with MD remain asymptomatic, 2-4% may develop complications. The most common symptom is painless rectal bleeding, typically caused by acid secretion from ectopic gastric tissue, which can ulcerate the mucosa and result in bleeding. Other potential manifestations include bowel obstruction, leakage of small bowel contents through the umbilicus (from a patent omphalomesenteric duct), or a purulent discharge at the umbilicus (umbilical sinus) [8,9,14].

In this specific case, there was no prior knowledge that the patient had an MD, as there was no evidence of it in imaging and the patient had no history of abdominal surgeries and was asymptomatic until this episode. It is likely that a fibrous band caused the internal hernia, leading to the obstruction. The prompt approach to the patient's condition resulted in a successful outcome, as there were already signs of local ischemia. Luckily, only the segment containing the MD had ischemic signs, and only that segment needed to be removed allowing an ileo-ileal anastomosis.

Symptomatic MD requires surgical resection; however, since MD can be an incidental finding during surgery, special awareness of management options and knowledge to support these options is crucial to providing the best care for patients. In individuals under 18 years of age, surgical resection of incidentally found MD during abdominal exploration is recommended because of the higher risk of complications of MD compared to adults [15].

For male adults under 50, surgical resection of incidentally found MD is recommended due to a higher risk of complications. For females under 50, resection is advised only if the diverticulum is over 2 cm, has a fibrous band, or shows palpable abnormalities, as these factors indicate increased risk [8,16].

For patients aged 50 and above, surgical removal of MD is advised only when abnormalities, such as ectopic tissue, hardened areas, or ulcers, are detectable as these can lead to bleeding. Additionally, while the likelihood of cancer within the diverticulum is higher in older adults compared to younger individuals, it remains rare overall, and the risks associated with surgery are greater in this age group [17].

Regarding surgical techniques, MD can be removed through diverticulectomy or segmental resection with anastomosis. Segmental resection is indicated if diverticulectomy risks narrowing the bowel lumen if there is a palpable abnormality at the base, or if the diverticulum has a wide neck (> 2 cm). Broad-based, short diverticula with a height-to-diameter ratio < 2 that require resection are better managed with small bowel resection to avoid leaving ectopic tissue at the base. If there are bleeding symptoms or a documented ulcer from ectopic gastric tissue, segmental resection should be performed [18,19].

## Conclusions

MD, while often asymptomatic, can lead to significant complications such as small bowel obstruction, gastrointestinal bleeding, or infection. In this case report, a previously undiagnosed MD presented as an acute small bowel obstruction, underscoring the importance of considering MD in differential diagnoses, especially in patients with no prior history of abdominal surgeries. Although the risk of complications increases with age, careful monitoring and surgical intervention when indicated remain crucial for preventing severe outcomes. Given the heterogeneity of MD presentations, clinicians must maintain a high index of suspicion for this condition, particularly when unusual abdominal symptoms arise, and be prepared to tailor surgical decisions based on the patient’s risk factors and presentation.

## References

[1] McLin VA, Henning SJ, Jamrich M (2009). The role of the visceral mesoderm in the development of the gastrointestinal tract. Gastroenterology.

[2] Sagar J, Kumar V, Shah DK (2006). Meckel's diverticulum: a systematic review. J R Soc Med.

[3] Simms MH, Corkery JJ (1980). Meckel's diverticulum: its association with congenital malformation and the significance of atypical morphology. Br J Surg.

[4] Yahchouchy EK, Marano AF, Etienne JC, Fingerhut AL (2001). Meckel's diverticulum. J Am Coll Surg.

[5] Hansen CC, Søreide K (2018). Systematic review of epidemiology, presentation, and management of Meckel's diverticulum in the 21st century. Medicine (Baltimore).

[6] Ueberrueck T, Meyer L, Koch A, Hinkel M, Kube R, Gastinger I (2005). The significance of Meckel's diverticulum in appendicitis — a retrospective analysis of 233 cases. World J Surg.

[7] Soltero MJ, Bill AH (1976). The natural history of Meckel’s diverticulum and its relation to incidental removal. A study of 202 cases of diseased Meckel’s diverticulum found in King County, Washington, over a fifteen year period. Am J Surg.

[8] Park JJ, Wolff BG, Tollefson MK, Walsh EE, Larson DR (2005). Meckel diverticulum: the Mayo Clinic experience with 1476 patients (1950-2002). Ann Surg.

[9] Dumper J, Mackenzie S, Mitchell P, Sutherland F, Quan ML, Mew D (2006). Complications of Meckel's diverticula in adults. Can J Surg.

[10] Bagade S, Khanna G (2015). Imaging of omphalomesenteric duct remnants and related pathologies in children. Curr Probl Diagn Radiol.

[11] Arnold JF, Pellicane JV (1997). Meckel's diverticulum: a ten-year experience. Am Surg.

[12] Shelat VG, Kelvin Li K, Rao A, Sze Guan T (2011). Meckel's diverticulitis causing small bowel obstruction by a novel mechanism. Clin Pract.

[13] Elsayes KM, Menias CO, Harvin HJ, Francis IR (2007). Imaging manifestations of Meckel's diverticulum. AJR Am J Roentgenol.

[14] An J, Zabbo CP (2023). Meckel diverticulum. StatPearls.

[15] Onen A, Ciğdem MK, Oztürk H, Otçu S, Dokucu AI (2003). When to resect and when not to resect an asymptomatic Meckel's diverticulum: an ongoing challenge. Pediatr Surg Int.

[16] Mackey WC, Dineen P (1983). A fifty year experience with Meckel's diverticulum. Surg Gynecol Obstet.

[17] Zani A, Eaton S, Rees CM, Pierro A (2008). Incidentally detected Meckel diverticulum: to resect or not to resect?. Ann Surg.

[18] Rivas H, Cacchione RN, Allen JW (2003). Laparoscopic management of Meckel's diverticulum in adults. Surg Endosc.

[19] Varcoe RL, Wong SW, Taylor CF, Newstead GL (2004). Diverticulectomy is inadequate treatment for short Meckel's diverticulum with heterotopic mucosa. ANZ J Surg.

